# Sex- and site-specific differences in colorectal cancer risk among people with type 2 diabetes

**DOI:** 10.1007/s00384-018-3191-7

**Published:** 2018-11-12

**Authors:** Jetty A. Overbeek, Josephina G. Kuiper, Amber A. W. A. van der Heijden, Mariette Labots, Ulrike Haug, Ron M. C. Herings, Giel Nijpels

**Affiliations:** 1 Department of General Practice and Elderly Care Medicine, Amsterdam Public Health Research Institute, VU University Medical Centre, Van der Boechorststraat 7, 1081 BT Amsterdam, Netherlands; 2 PHARMO Institute for Drug Outcomes Research, Utrecht, Netherlands; 3 Department of Public Health, Erasmus University Medical Centre, Rotterdam, Netherlands; 4 Department of Medical Oncology, VU University Medical Center, Amsterdam, Netherlands; 5 Department of Clinical Epidemiology, Leibniz Institute for Prevention Research and Epidemiology – BIPS, Bremen, Germany; 6 Faculty of Human and Health Sciences, University of Bremen, Bremen, Germany; 7 Department of Epidemiology & Biostatistics, VU University Medical Center, Amsterdam, Netherlands

**Keywords:** Type 2 diabetes, Sidedness, Proximal colon cancer, Distal colon cancer, Right-sided colon cancer, Left-sided colon cancer

## Abstract

**Purpose:**

The prevalence of colorectal cancer is higher among patients with type 2 diabetes mellitus (T2D) than among patients without diabetes. Furthermore, men are at higher risk for developing colorectal cancer than women in the general population and also subsite-specific risks differ per sex. The aim was to evaluate the impact of T2D on these associations.

**Methods:**

A population-based matched cohort study was performed using data from the PHARMO Database Network. Patients with T2D were selected and matched (1:4) to diabetes free controls. Cox proportional hazards models were used to estimate hazard ratios (HRs) for CRC and its subsites. HRs were determined per sex and adjusted for age and socioeconomic status. The ratio of distal versus proximal colon cancer was calculated for people with T2D and controls per sex and stratified by age.

**Results:**

Over 55,000 people with T2D were matched to > 215,000 diabetes free controls. Men and women with T2D were 1.3 times more likely to develop colorectal cancer compared to controls. Men with T2D were at higher risk to develop distal colon cancer (hazard ratio (95% confidence interval), 1.42 (1.08–1.88)), and women with T2D were at higher risk for developing proximal colon cancer (hazard ratio (95% confidence interval), 1.58 (1.13–2.19)). For rectal cancer, no statistically significant risk was observed for both men and women.

**Conclusions:**

Sex-specific screening strategies and prevention protocols should be considered for people with T2D. More tailored screening strategies may optimize the effectiveness of colorectal cancer screening in terms of reducing incidence and mortality.

**Electronic supplementary material:**

The online version of this article (10.1007/s00384-018-3191-7) contains supplementary material, which is available to authorized users.

## Introduction

Type 2 diabetes mellitus (T2D) and colorectal cancer (CRC) are increasing health problems. Currently, CRC is the third most common cancer worldwide and the second most common cancer in Europe [1]. The number of people with CRC is expected to increase due to demographic changes, obesity, and lack of physical activity. Also, the prevalence of T2D is increasing worldwide [2, 3]. In the Netherlands, the prevalence of T2D more than doubled between 1999 and 2014, mainly due to demographic changes, but probably also due to overweight and screening initiatives [4].

Regardless of T2D, CRC incidence, prevalence, and mortality are higher among men than women [5, 6]. However, women aged ≥ 55 years are more often diagnosed with proximal (right-sided) CRC [7], which is associated with more aggressive form of neoplasia than distal (left-sided) CRC [8]. Among these reasons, sex-specific screening strategies have been proposed [9].

Several observational studies have demonstrated an increased risk of CRC in people with T2D [10, 11]. Several mechanisms have been proposed to explain the higher prevalence of CRC in people with hyperglycemia, such as hyperglycemia in itself, hyperinsulinemia, which leads to increased insulin-like growth factor (IGF) levels, and insulin resistance [12].

Some reviews and meta-analyses regarding the association between T2D and CRC reported a higher risk of CRC among women with T2D (compared to their disease-free controls) [13] than among men with T2D (compared to their disease-free controls) [14], while others concluded that the risk among people with T2D compared to people without T2D is regardless of sex [15–18].

Sex-specific differences in risk of anatomical subsites of CRC in people with T2D are less studied. As people with T2D already undergo health check-ups regularly, it is important to know whether sex-specific screening strategies would also be necessary for people with T2D. Therefore, the aim of the current study was to evaluate the sex-specific risk of subsites of CRC in people with T2D compared to people without diabetes in a population-based cohort. In this study, the unique linkage between the General Practitioner (GP) Database of the PHARMO Database Network and the Netherlands Cancer Registry (NCR) was used, creating a comprehensive large database with detailed and high-quality data on cancer and T2D.

## Material and Methods

### Data sources

Data for this cohort study were obtained from the GP Database of the PHARMO Database Network [19] and the NCR. The GP Database comprises data from electronic patient records registered by GPs. The records include information on diagnoses and symptoms, laboratory test results, referrals to specialists, and healthcare product/drug prescriptions. Currently, the GP Database covers a catchment area of approximately 3.5 million inhabitants. Recently, the GP Database was linked to the NCR on a patient-level. The NCR is maintained by the Netherlands Comprehensive Cancer Organization (IKNL) [20] and contains information on newly diagnosed patients with cancer, coded according to the WHO International Classification of Diseases for Oncology (ICD-O-3). The NCR is notified, on a daily basis, for new patients with cancer by pathology departments, general hospitals, and radiotherapy institutes. The construct of the record linkage method is described elsewhere [21]. The privacy committees of the NCR and the PHARMO Institute approved this study.

### Study population

From PHARMO’s GP Database, all people diagnosed with T2D between 2006 and 2014 were selected. T2D was defined as a recorded episode for T2D or ≥ 2 prescriptions of a blood glucose–lowering drug, excluding insulin, within a 6-month period at any time in the available medication records. The date of the first recorded episode for T2D, the second prescription, or the first examination regarding diabetes, whichever occurred first, was defined as the index date. People with another type of diabetes, using insulin prior to index date, < 40 years of age at index date, or having a history of cancer were excluded (see Supplementary Table S1 for codes used for exclusion criteria). Patients with < 12 months of continuous enrolment prior to index date were excluded as well, in order to ensure newly diagnosed people with T2D.

People with T2D were randomly matched (1 up to 4) to controls on sex, year of birth (± 2 years), GP practice, and start year of enrolment in the database. Matched controls received the same index date as their matched cases. Controls who had a history of diabetes, were < 40 years of age at index date, had < 12 months of continuous enrolment prior to index date, or had a history of cancer were excluded. Furthermore, controls had to be alive and known in the GP Database at index date and could not be matched to themselves or more than once.

All people with T2D and matched controls were followed from index date until diagnosis of CRC, diagnosis of (another type of) diabetes, end of database registration (i.e., patient moves out of the catchment area), death, or end of study period (December 31, 2014), whichever occurred first.

### Characteristics

For all included people, the following was determined at index date: age, socioeconomic status (SES), available history and follow-up in the database, and year of index date. Furthermore, the use of aspirin, non-aspirin non-steroidal anti-inflammatory drugs (NSAIDs), statins, antihypertensives, and hormone replacement therapy (HRT) was determined in the year prior to index date (see Supplementary Table S1 for ATC codes). SES was derived from Statistics Netherlands [22], which based SES on salary per 6-digit zip code determined in December 2008.

### Outcome

During follow-up, the occurrence of the initial diagnosis of primary, localized (or non-metastatic) CRC was obtained from the NCR and used as outcome in the analyses. Proximal colon cancers included malignant neoplasms of cecum, appendix, ascending colon, hepatic flexure, and transverse colon. Distal colon cancers included malignant neoplasms of splenic flexure, descending colon, and sigmoid colon. Rectal cancer included malignant neoplasm of rectum. Malignant neoplasm of overlapping sites of colon, unspecified sites of colon, and rectosigmoid junction were included when analyzing overall CRC.

### Statistical methods

Characteristics of all included people were reported descriptively. Differences in characteristics between men and women with T2D were compared with men and women without diabetes and assessed using chi-square tests for categorical variables and ANOVA tests for continuous variables.

Unadjusted incidence rates (IRs) for CRC were determined by dividing the total number of events by the total number of patient years at risk (summed number of years of follow-up). To generate hazard ratios (HR) and their corresponding 95% confidence intervals (CI), Cox proportional hazards model, adjusted for age, SES, and drugs known to (potentially) influence risk of CRC (aspirin, non-aspirin NSAIDs, statins, antihypertensives, and HRT) were used. The analyses were stratified according to three categories regarding anatomic subsites (proximal colon, distal colon, and rectum) and risk estimates were also calculated for each subsite separately.

As several studies have reported a shift of CRC localization by age [7], it was determined whether the same trend was observed among people with T2D. The number of distal (including rectal) colon cancers was divided by the number of proximal colon cancers to calculate the ratio of distal versus proximal colon cancer. This ratio was calculated for people with T2D and no diabetes per sex and was stratified by age (50–69 and ≥ 70 years) at index date.

All data were analyzed using SAS programs organized within SAS Enterprise Guide version 4.3 (SAS Institute Inc., Cary, NC, USA) and conducted under Windows using SAS version 9.2.

#### Sensitivity analyses

Reported associations in observational studies can be affected by detection (protopathic) bias, i.e., an increased odds of detecting cancer shortly after the onset of diabetes [10]. In order to explore the extent of detection bias, the risk of (anatomic subsites of) CRC was stratified by follow-up period (0–91 days, > 91–182 days, > 182–365 days, and > 365 days). Per follow-up period, people with the date of CRC not within the follow-up period were censored. Only follow-up up to the end of that specific follow-up period, end of follow-up, or date of CRC, whichever occurred first, was used to calculate the total number of patient years at risk.

## Results

### Patient characteristics

After applying all in- and exclusion criteria, 29,696 men and 25,349 women with T2D were included and matched to 116,570 and 99,437 diabetes free controls, respectively (see Supplementary Fig. S1). Mean age at baseline was 62.1 years among men and 64.9 years among women. Baseline characteristics, such as age, SES, history in the database, and year of index date, were similar between people with T2D and people without diabetes. Available follow-up in the database was longer among cases compared to controls. Furthermore, people with T2D more often used aspirin, non-aspirin NSAIDs, statins, and antihypertensives compared to the matched people without diabetes (see Table 1).

**Table 1:** General characteristics of people with T2D and no diabetes

	Men			Women		
	T2D *N* = 29,696 n (%)	No diabetes *N* = 116,570 n (%)	*p* value	T2D *N* = 25,349 n (%)	No diabetes *N* = 99,437 n (%)	*p* value
Age (years), mean ± SD	62.1 ± 10.9	62.0 ± 10.9	0.06	64.9 ± 12.2	64.8 ± 12.2	0.10
SES			0.99			0.99
Low	7460 (25)	29,247 (25)		6570 (26)	25,722 (26)	
Normal	9481 (32)	37,217 (32)		8281 (33)	32,507 (33)	
High	12,755 (43)	50,106 (43)		10,498 (41)	41,208 (41)	
Patient history in database (years)			0.24			0.28
Mean ± SD	4.0 ± 2.1	4.1 ± 2.1		3.9 ± 2.1	4.0 ± 2.1	
Median (IQR)	3.8 (2.2–5.6)	3.8 (2.2–5.7)	.24	3.6 (2.2–5.4)	3.6 (2.2–5.5)	
Follow-up in database (years)			< .0001			< .0001
Mean ± SD	3.7 ± 2.2	3.5 ± 2.2		3.8 ± 2.2	3.7 ± 2.2	
Median (IQR)	3.7 (1.9–5.5)	3.4 (1.7–5.3)		3.8 (2.0–5.6)	3.7 (1.8–5.5)	
Year of index date			0.60			0.53
2007–2008	6205 (21)	24,691 (21)		5644 (22)	22,477 (23)	
2009–2010	8528 (29)	33,612 (29)		7579 (30)	29,869 (30)	
2011–2012	8059 (27)	31,447 (27)		6598 (26)	25,688 (26)	
2013–2014	6904 (23)	26,820 (23)		5528 (22)	21,403 (22)	
Co-medication^a^						
Aspirin	4972 (17)	12,303 (11)	< .0001	3236 (13)	8197 (8)	< .0001
Non-aspirin NSAIDs	6999 (24)	22,274 (19)	< .0001	6594 (26)	22,092 (22)	< .0001
Statins	11,626 (39)	22,828 (20)	< .0001	8800 (35)	15,712 (16)	< .0001
Antihypertensives	13,208 (44)	28,925 (25)	< .0001	13,469 (53)	30,609 (31)	< .0001
HRT	–	–		814 (3)	3656 (4)	< .01

*SD* standard deviation, *IQR* interquartile range, *NSAIDs* non-steroidal anti-inflammatory drugs, *HRT* hormone replacement therapy,

^a^Determined in the year prior to index date

### Colorectal cancer

**Fig. 1:**
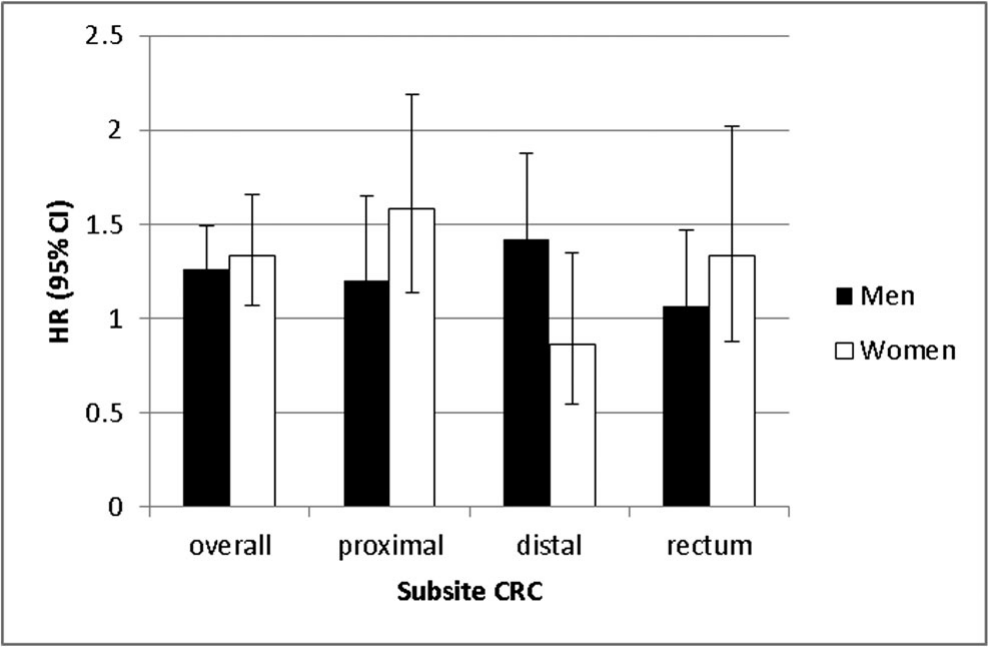
Difference between men and women in subsite CRC among people with T2D compared to people without diabetes

Figure 1 and Table 2 present the subsite-specific rates of CRC among men and women with T2D and their matched controls. Supplementary Table S2 presents the number of CRC events and person years at risk among men and women with T2D and no diabetes. Overall, both men and women with T2D were 1.3 times more likely to develop CRC compared to their controls without diabetes. However, differences regarding subsite-specific risks were observed between the sexes. Compared to diabetes free controls, men with T2D were at higher risk to develop distal colon cancer (HR (95% CI), 1.42 (1.08–1.88)) than women with T2D (HR (95% CI), 0.86 (0.55–1.35)). The same trend was observed in the anatomical subsites of distal colon cancer, except for cancer of the splenic flexure. Again, compared to controls without diabetes, women with T2D were at higher risk to develop proximal colon cancer (HR (95% CI), 1.58 (1.13–2.19)) than men with T2D (HR (95% CI), 1.20 (0.87–1.65)). This difference was also observed for all subsites of proximal colon cancer, although not always statistically significant. Women with T2D had a higher risk to develop rectal cancer than men with T2D compared to diabetes free controls, but the risk in both men and women was not statistically significant (HR (95% CI) for men is 1.06 (0.77–1.47) and 1.33 (0.88–2.02) for women).

**Table 2:** Incident rates and hazard ratios of subsites of CRC among men and women with T2D and no diabetes

	Men				Women			
	T2D	No diabetes	T2DM vs. no diabetes		T2D	No diabetes	T2DM vs. no diabetes	
	IR (95% CI)	IR (95% CI)	HR^a^	(95% CI)	IR (95% CI)	IR (95% CI)	HR*	(95% CI)
Colon and rectum	1.66 (1.43–1.92)	1.31 (1.20–1.43)	1.26	(1.06–1.49)	1.21 (1.00–1.45)	0.95 (0.85–1.05)	1.33	(1.07–1.65)
Proximal	0.47 (0.35–0.61)	0.39 (0.33–0.45)	1.20	(0.87–1.65)	0.55 (0.41–0.72)	0.37 (0.31–0.44)	1.58	(1.13–2.19)
Cecum	0.17 (0.10–0.27)	0.17 (0.13–0.22)	0.96	(0.57–1.62)	0.23 (0.14–0.34)	0.17 (0.13–0.22)	1.37	(0.83–2.27)
Appendix	–	–	–	–	–	–	–	–
Ascending colon	0.21 (0.13–0.31)	0.10 (0.08–0.14)	2.12	(1.26–3.59)	0.18 (0.10–0.28)	0.10 (0.07–0.13)	1.98	(1.09–3.61)
Hepatic flexure	0.05 (0.02–0.12)	0.03 (0.01–0.05)	2.31	(0.83–6.48)	0.08 (0.04–0.16)	0.04 (0.02–0.07)	2.21	(0.92–5.32)
Transverse colon	0.04 (0.01–0.09)	0.08 (0.05–0.11)	0.41	(0.14–1.18)	0.06 (0.02–0.14)	0.05 (0.03–0.08)	1.22	(0.47–3.13)
Distal	0.66 (0.52–0.83)	0.46 (0.40–0.54)	1.42	(1.08–1.88)	0.26 (0.17–0.38)	0.29 (0.24–0.35)	0.86	(0.55–1.35)
Splenic flexure	0.01 (0.00–0.05)	0.02 (0.01–0.04)	0.30	(0.04–2.37)	0.06 (0.02–0.14)	0.03 (0.01–0.05)	2.39	(0.82–6.93)
Descending colon	0.07 (0.03–0.14)	0.03 (0.01–0.05)	2.56	(1.00–6.53)	0.02 (0.00–0.07)	0.03 (0.01–0.05)	0.60	(0.13–2.85)
Sigmoid colon	0.58 (0.44–0.74)	0.41 (0.35–0.48)	1.42	(1.05–1.91)	0.18 (0.10–0.28)	0.23 (0.19–0.29)	0.72	(0.42–1.24)
Rectum	0.46 (0.34–0.60)	0.42 (0.36–0.49)	1.06	(0.77–1.47)	0.33 (0.23–0.47)	0.26 (0.21–0.32)	1.33	(0.88–2.02)

^a^Adjusted for age, SES, and the use of aspirin, non-aspirin NSAIDs, statins, antihypertensives, and HRT in the year prior to index date

**Fig. 2:**
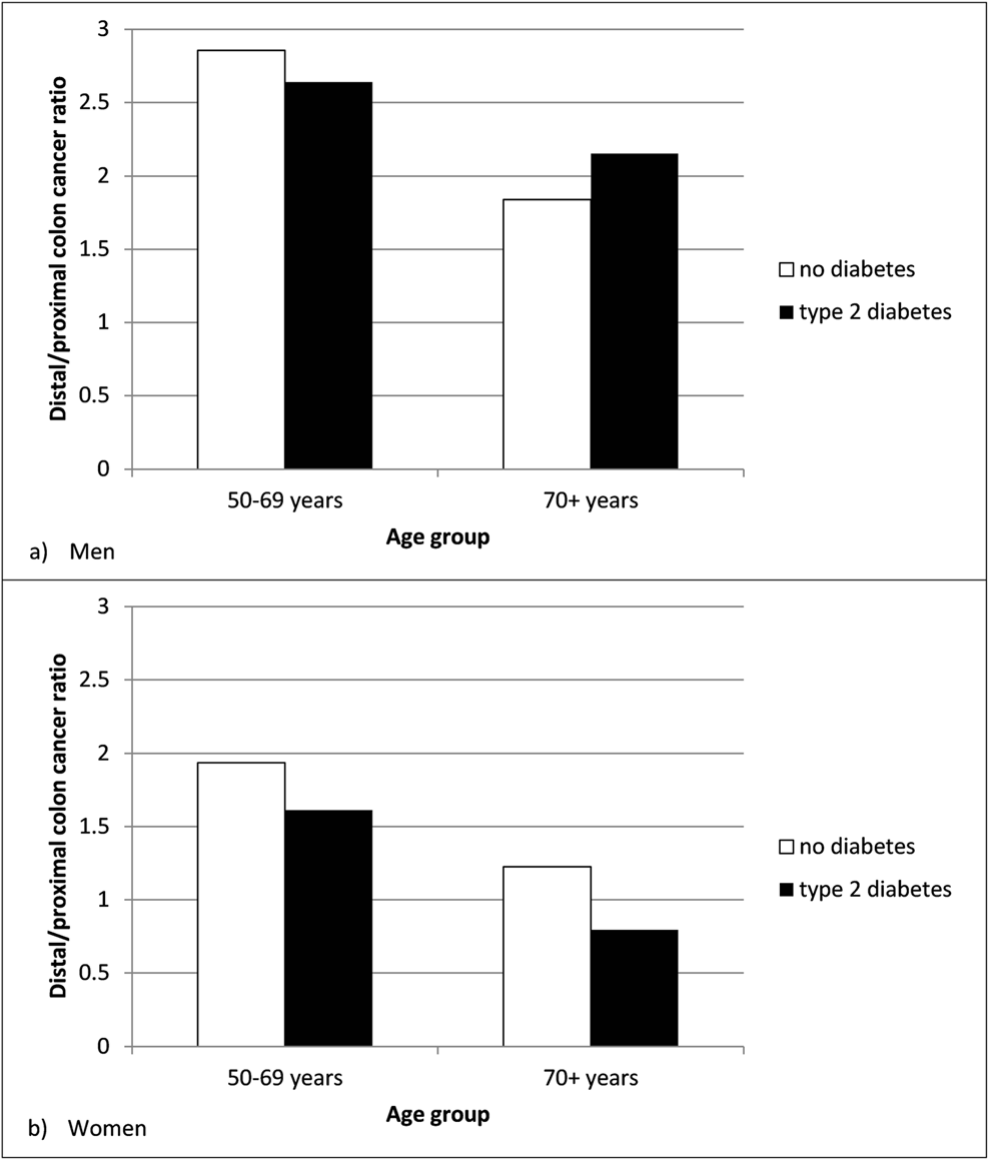
Ratio of distal (including rectal)/proximal colon cancer for people with T2D and no diabetes by age group among men (**a**) and women (**b**)

Figure 2 presents the ratio of distal (including rectal) versus proximal colon cancer stratified by T2D status in men (Fig. 2a) and women (Fig. 2b). As presented in Fig. 2a, distal colon cancer is more frequent than proximal colon cancer (i.e., ratio > 1) in men with T2D and no diabetes. The same is observed in men aged ≥ 70 years; however the ratio is lower than for men aged 50–69 years. As shown in Fig. 2b the ratio among women is also above 1 (i.e., more distal than proximal colon cancers), except for women with T2D aged ≥ 70 years, i.e., these women are more likely to be diagnosed with proximal colon cancer than with distal colon cancer. Generally, the ratio was lower for women than for men, i.e., irrespective of age and T2D status.

#### Sensitivity analysis

To account for potential detection bias, risk of (anatomical subsites of) CRC was stratified by follow-up period (Supplementary Fig. S2). When considering a 1-year lag period, the risk of overall CRC and its subsites was similar than the risk calculated without a lag period; only the risk of distal colon cancer became slightly lower through consideration of a 1-year lag period. The differences in risk between men and women remained after applying the lag period.

## Discussion and conclusion

In this population-based cohort study among more than 270.000 people, we observed a similarly increased risk of CRC among men and women with T2D compared to diabetes free controls. However, differences regarding the location of the CRC were observed. Compared to diabetes free controls, men with T2D had a higher increased risk of distal colon cancer than women with T2D, and women with T2D had a higher increased risk of proximal colon cancer than men with T2D. These findings remained after applying a 1-year lag period to account for detection bias. Furthermore, women with T2D aged ≥ 70 years are more likely to develop proximal rather than distal colon cancer.

The overall risk of CRC observed in our study among men and women with T2D compared to men and women without diabetes is in line with previously published papers [13, 15–17]. Several epidemiological studies presented separate risks for proximal and distal colon cancer by sex among people with T2D [11, 23–27], but also regardless of diabetes [9, 28].

The majority of these studies among people with T2D showed a higher increased risk of *proximal* colon cancer in women with T2D (HR ranging from 1.6 to 1.8) than men with T2D (HR ranging from 1.4 to 1.6) compared to diabetes free controls, which is consistent with our finding [11, 23–25, 27]. One study [26] found, compared to diabetes free controls, a higher increased risk of overall, proximal, and distal CRC among men than women. However, men were almost twice more likely to be classified as current or former cigarette smoker and they believe effect modification from cigarette smoking status appeared to have contributed to the difference in risk observed by sex.

Our finding that, compared to diabetes free controls, men are at higher risk to develop *distal* colon cancer than women appears to be consistent with existing literature [11, 23–27]. Four of these studies [23, 25–27] found higher risks of distal colon cancer among men (HR ranging from 1.3 to 2.1) than women (HR ranging from 0.7 to 2.0). The other two studies [11, 24] found a higher risk of distal colon cancer among women than among men, but the results of these studies were not statistically significant.

For *rectal* cancer, studies regarding its sex-specific association with T2D are less consistent. One meta-analysis [29] found a statistically significant association between diabetes and rectal cancer for men (HR (95% CI), 1.22 (1.07–1.40)), but not for women (1.09 (0.99–1.19)). Two studies [25, 27] found a statistically significant increased risk of rectal cancer for women, which was higher than the non-statistically significant increased risk for men. Other cohort studies [10, 11, 23] did not find a statistically significant association for men or women, which was similar to our study.

In the general population, differences in the association between sex and anatomic subsites of CRC have been explained by the fact that the proximal colon, distal colon, and rectum have different embryological origins [30, 31]. In addition, hormonal factors, (epi) genetic differences, dietary factors, and structural factors have been proposed [9]. Furthermore, tumor suppressor genes, point mutations, genetic instability, and responses of cells to growth stimulating hormones, such as IGF, may differ by CRC subsite [11]. As it is hypothesized that both diabetes and CRC involve over-expression of both the insulin and IGF receptors [32], this potentially even more complicates the association. Epidemiological evidence also links hyperinsulinemia to changes in sex steroids [33]. Sex differences in relation to certain risk factors may modify risk for tumor development, such as alcohol consumption, smoking, and red meat consumption [31, 34]. All taken together, it is likely that all these factors interact and act differently at various locations of the colorectum.

The results from our study suggest that sex-specific screening strategies are even more important among people with diabetes. While women without diabetes are known to present with proximal colon cancer more often than men, we found that women with T2D have an even higher increased risk of proximal colon cancer than women without diabetes. Furthermore, women with T2D aged ≥ 70 years were even more likely to be diagnosed with a proximal colon cancer than with a distal colon cancer. More attention should be paid to the adherence to colonoscopy screening in this risk group, being better suited to detect lesions in the proximal colon than other screening options.

Some limitations of this observational study should be mentioned. First, possible important confounders, such as obesity, smoking status, physical inactivity and nutritional intake could not be corrected for in our analyses. However, previous epidemiological studies, presenting both crude and adjusted risks, showed that adjusting for these factors only slightly attenuated the risk. Second, only patients with a GP recorded diagnosis or treated with blood glucose–lowering drugs were included. Therefore, misclassification of T2D could have occurred as some patients are undiagnosed [35]. Furthermore, detection bias is a common phenomenon. People with T2D are more likely to be diagnosed with cancer shortly after the onset of diabetes as compared to people without diabetes. By applying a one-year lag period, we aimed to exclude this bias, although the right lag period to exclude detection bias remains unknown [10]. Finally, as only outcome information regarding CRC was available, other forms of cancer as competing outcomes could not be taken into account. However, this will not affect the differences observed between people with T2D compared to people without diabetes.

Overall, this is the first study using the linkage between the NCR and the GP Database of the PHARMO Database Network for the association between T2D and sex- and site-specific difference in CRC risk. By linking these databases, a unique cohort was created taking advantage of the high-quality data on cancer and detailed information regarding T2D. This linkage resulted, to our knowledge, to one of the largest, detailed cohorts of people with T2D in which the incidence of subsite-specific CRC could be studied. An advantage of using the GP Database for selecting people with T2D is including people with T2D not yet pharmacologically treated (i.e., also people treated with lifestyle interventions). The improved linkage gave us the opportunity to also analyze the association between T2D and anatomical subsites of CRC.

Furthermore, ascertainment of exposure was based on large and high-quality pharmaco-epidemiological databases, which is more reliable than self-reported questionnaires. Because of repeated information regarding exposure, patients’ follow-up could be ended when (another type of) diabetes was diagnosed (i.e., decreasing the likelihood of misclassification of T2D).

## Conclusion

Besides a similarly increased risk of CRC among men and women with T2D compared to diabetes free controls, we found a higher increased risk of proximal colon cancer among women with T2D than men with T2D and a higher increased risk of distal colon cancer among men with T2D than women with T2D, compared to diabetes free controls. Therefore, sex-specific screening and prevention protocols may be considered for people with T2D. More tailored screening strategies may optimize the effectiveness of CRC screening in terms of reducing CRC incidence and mortality and improving the quality of life. Furthermore, future studies investigating the association between T2D and CRC should include sex-specific and subsite-specific analyses.

## Electronic supplementary material

ESM 1 (DOCX 48 kb)
