# Supplementary material for: Sex- and site-specific differences in colorectal cancer risk among people with type 2 diabetes
Source: Int J Colorectal Dis. 2018 Nov 12;34(2):269–76. doi: 10.1007/s00384-018-3191-7 (PMC6331739; doi:10.1007/s00384-018-3191-7)
Supplement: Supplementary file 1 — (DOCX 48 kb) [file 384_2018_3191_MOESM1_ESM.docx]

### Supplementary Table S1: Codes used for exclusion and co-medication

|  | **Coding system** | **Code** | **Description** |
| --- | --- | --- | --- |
| **Exclusion criteria** |  |  |  |
| Diagnosis of another type of diabetes than type 2 diabetes | ICPC | T90.01 | Type 1 Diabetes Mellitus |
|  | Free text | - | Secondary diabetes |
|  | Free text | - | MODY |
| Use of insulin | ATC | A10A | Insulins and analogues |
| Cancer diagnosis | ATC | L01 | Antineoplastic agents |
|  | ATC | L02B | Hormone antagonists and related agents |
|  | ATC | L03AA | Colony stimulating factors (immunostimulants) |
|  | ICPC | A79 | Malignancy NOS |
|  | ICPC | B72-B74 | Hodgkin’s disease/lymphoma/leukaemia/other malignant neoplasm blood |
|  | ICPC | D74-D77 | Malignant neoplasm stomach/colon/rectum/pancreas/digest other/digest NOS |
|  | ICPC | N74 | Malignant neoplasm nervous system |
|  | ICPC | R84-R85 | Malignant neoplasm bronchus/lung/respiratory, other |
|  | ICPC | S77, excl. S77.01 | Malignant neoplasm of skin, excluding ‘in situ’ and ‘basal cell carcinoma’ |
|  | ICPC | T71 | Malignant neoplasm thyroid |
|  | ICPC | U75-U77 | Malignant neoplasm of kidney/bladder/urinary other |
|  | ICPC | W72 | Malignant neoplasm relate to pregnancy |
|  | ICPC | X75-X77 | Malignant neoplasm cervix/breast female/genital other (f) |
|  | ICPC | Y77-Y78 | Malignant neoplasm prostate/genital other (m) |
|  | ICD-O-3 | C18-C20 | Malignant neoplasm of colon/rectosigmoid junction/rectum |
| **Co-medication** |  |  |  |
| Aspirin | ATC | B01AC06 | Acetylsalicylic acid |
|  |  | B01AC30 | Clopidogrel/acetylsalicylic acid |
|  |  | B01AC56 | Acetylsalicylic acid/esomeprazole |
|  |  | N02BA01 | Acetylsalicylic acid |
|  |  | N02BA51 | Acetylsalicylic acid/ascorbic acid, acetylsalicylic acid/metoclopramide |
| Non-aspirin NSAIDs | ATC | M01A, excluding M01AX | Antiinflammatory and antirheumatic products, non-steroids |
| Statins | ATC | C10AA | HMG CoA reductase inhibitors |
| Antihypertensives | ATC | C02 | Antihypertensives |
|  | ATC | C03 | Diuretics |
|  | ATC | C07 | Beta blocking agents |
|  | ATC | C08 | Calcium channel blockers |
|  | ATC | C09 | Agents acting on the renin-angiotensin system |
| HRT | ATC | G03C, G03F | Estrogens, progestogens and estrogens in combination |

### Supplementary Table S2: Number of CRC events and person years at risk among men and women with type 2 diabetes and without diabetes

|  | **Men** | | | | **Women** | | | | |
| --- | --- | --- | --- | --- | --- | --- | --- | --- | --- |
|  | **type 2 diabetes** | | **no diabetes** | | **type 2 diabetes** | | | **no diabetes** | |
|  | **N_event_** | **PY at risk** | **N_event_** | **PY at risk** | **N_event_** | **PY at risk** | | **N_event_** | **PY at risk** |
| **Colon and rectum** | 184 | 110,785 | 539 | 410,576 | 117 | 96,446 | | 347 | 365,984 |
| Proximal | 52 | 111,034 | 160 | 411,402 | 53 | 96,583 | | 137 | 366,512 |
| Coecum | 19 | 111,088 | 71 | 411,608 | 22 | 96,627 | | 63 | 366,676 |
| Appendix | 0 | 111,118 | 4 | 411,737 | 0 | 96,657 | | 3 | 366,773 |
| Ascending colon | 23 | 111,082 | 43 | 411,659 | 17 | 96,630 | | 35 | 366,693 |
| Hepatic flexure | 6 | 111,106 | 11 | 411,718 | 8 | 96,646 | | 16 | 366,738 |
| Transverse colon | 4 | 111,111 | 31 | 411,660 | 6 | 96,650 | | 20 | 366,742 |
| Distal | 73 | 110,984 | 191 | 411,364 | 25 | 96,600 | | 106 | 366,521 |
| Splenic flexure | 1 | 111,117 | 10 | 411,738 | 6 | 96,644 | | 10 | 366,760 |
| Descending colon | 8 | 111,107 | 11 | 411,717 | 2 | 96,656 | | 10 | 366,756 |
| Sigmoid colon | 64 | 110,995 | 170 | 411,399 | 17 | 96,613 | | 86 | 366,559 |
| Rectum | 51 | 111,020 | 173 | 411,331 | 32 | 96,592 | 97 | | 366,521 |

*Adjusted for age, SES and the use of aspirin, non-aspirin NSAIDs, statins, antihypertensives, and HRT in the year prior to index date.

| People with type 2 diabetes registered in PHARMO’s GP database between 2006 - 2014  N = 132,111 | | | | | | | | |
| --- | --- | --- | --- | --- | --- | --- | --- | --- |
|  |  | | | | | | | |
|  | \| **Exclusions** \| **N =** \| **76,125** \| \| --- \| --- \| --- \| \| - another type of diabetes \| n = \| 3,905 \| \| - insulin prior to index date \| n = \| 12,307 \| \| - <40 years of age at index date \| n = \| 4,764 \| \| - a cancer diagnosis prior to index date \| n = \| 4,226 \| \| - <12 months of continuous enrolment prior to index date \| n = \| 64,479 \| | | | | | | | |
|  |  | | | | | | | |
| People with type 2 diabetes between Jan 1, 2006 - Dec 31, 2014  N = 55,986 | | | | | | | | |
|  | | | | | | | | |
| Matched cohort | | | | | | | | |
| People with type 2 diabetes  N = 55,045 | | | | | People without diabetes  N = 216,007 | | | |
|  | |  |  |  | |  |  |  |
| Men with  type 2 diabetes  N = 29,696 | |  | Women with  type 2 diabetes  N = 25,349 |  | | Men without diabetes  N = 116,570 |  | Women without diabetes  N = 99,437 |

### Supplementary Figure S1: Flow chart of patient selection

| a) Overall CRC | b) Proximal colon cancer |
| --- | --- |
| c) Distal colon cancer | d) Rectal cancer |

### Supplementary Figure S2: Difference between men and women in subsite CRC among people with type 2 diabetes compared to people with no diabetes stratified by follow-up period. Black = men; white = women.
